# A CRITICAL REFLECTION ON AGING IN PLACE: INCLUDING END OF LIFE IN AGE-FRIENDLY POLICY

**DOI:** 10.1093/geroni/igad104.0391

**Published:** 2023-12-21

**Authors:** Patty Doran

**Affiliations:** The University of Manchester, Manchester, England, United Kingdom

## Abstract

By taking a critical perspective on the policy objective of ageing in place, this theoretical paper asks where are the end-of-life discussions in age-friendly policies? People do not like to think about “growing old”, even less so, perhaps, do they like to talk about end of life and dying. To overcome this, ageing policies have successively been focused on active, successful and healthy ageing. However, there have been increasing calls to examine ageing, and to support ageing in place, from a perspective that acknowledges the heterogeneity of the older population and the challenges faced by diverse groups of older people. The places in which we age, and our experiences of ageing, living and dying, are all influenced by social and political determinants. While separately there is increasing research highlighting inequity in both the ability of older adults to age in place and their experience at end-of-life, there is little literature that suggests how policy that supports ageing in place can also enable better experiences at end-of-life. By adopting a spatial justice lens to the concept of ageing in place, this paper first presents a critical reflection of the places in which we live and age. Second, the paper suggests there are overlapping challenges and opportunities in relation to supporting both ageing in place and end-of-life, therefore there is a need to include greater discussion of end-of-life and dying in age-friendly policy.

